# Coronary artery bypass graft surgery in patients on ticagrelor therapy is not associated with adverse perioperative outcomes

**DOI:** 10.1186/s13019-021-01521-y

**Published:** 2021-05-22

**Authors:** Sammer Diab, Mattan Arazi, Leonid Sternik, Ehud Raanani, Erez Kachel, Liza Grosman-Rimon, Amjad Shalabi, Offer Amir, Shemy Carasso

**Affiliations:** 1 Division of Cardiovascular Medicine and Surgery, Poriya Medical Center, Tiberias, Israel; 2 The Azrieli Faculty of Medicine in the Galilee, Bar-Ilan University, Safed, Israel; 3 Cardiac Surgery, Sheba Medical Center, Tel-Hashomer, Israel; 4 The Sackler Faculty of Medicine, Tel-Aviv University, Tel-Aviv, Israel; 5 Department of Cardiology, Hadassah Medical Center, Jerusalem, Israel; 6 Faculty of Medicine, Hebrew University of Jerusalem, Jerusalem, Israel

**Keywords:** Ticagrelor, Bleeding, Coronary artery bypass graft surgery, Dual anti platelet therapy, Coronary artery bypass graft, Acute coronary syndrome

## Abstract

**Background:**

Management of patients treated with Ticagrelor is challenging, as stopping Ticagrelor prior to coronary bypass graft surgery (CABG) may increase the risk of acute stent thrombosis. The aim of the study was to compare bleeding complications in patients treated with ticagrelor combined with acetylsalicylic acid (ASA) versus ASA alone until 1 day before surgery.

**Methods:**

Bleeding complications, defined as the composite of red blood cell transfusion ≥1000 ml, chest drainage ≥2000 ml, and bleeding requiring surgical re-exploration, were compared in 161 patients, with 101 on preoperative acetylsalicylic acid (ASA) alone (group A) and 65 on ticagrelor + ASA (group B).

**Results:**

There were no differences in bleeding complications between the two groups (26% vs. 27% in group A and B, respectively), with similar chest drainage in the first 24 h (569 ± 393 ml and 649 ± 427 ml, respectively).

**Conclusions:**

Continuing ticagrelor until coronary artery bypass surgery was not associated with increased bleeding complications, suggesting that continued management with ticagrelor until surgery may be safe.

## Introduction

Acute coronary syndrome (ACS) guidelines recommend that most patients receive dual antiplatelet therapy at the time of presentation to prevent recurrent ischemic events. Approximately 10% of ACS patients require coronary artery bypass grafting surgery (CABG) [1]. Excessive bleeding after cardiac surgery is associated with transfusion of blood products, which is associated with increased morbidity, mortality and high costs [2]. One important factor resulting in impaired hemostasis is preoperative use of antithrombotic medications such as platelet inhibitors and anticoagulants [3]. Discontinuation of antiplatelet therapy for a time frame prior to surgery to allow recovery of platelet function is warranted [4–7].

However, premature discontinuation of antiplatelet therapy in these settings has been associated with an increase in ischemic complications [8–11] and a reported mortality rate of 25–40% [12]. Indeed, ischemic events occur as high as 35.4% if antiplatelets are discontinued less than 30 days after placement of a coronary drug eluting stent, and decline to 11.7% if discontinued later but within the first 6 months, with a reported mortality rate of 25–40% [13]. Ticagrelor is an oral, reversibly binding and directly acting P2Y12 receptor antagonist that rapidly peaks within 1.5–3 h, has a rapid onset of antiplatelet effects, and half-life of 7 to 8 h. Furthermore, this reversibility may offer great flexibility for surgical procedures [14]. The optimal discontinuation time of P2Y12 antagonists before CABG is still poorly defined. Data regarding the true incidence of bleeding with shorter discontinuation periods of P2Y12 antagonists are lacking. Continuing ticagrelor to less than 2 days prior to surgery or at time of surgery was associated with a 3.5 times higher risk of platelet transfusions compared to patients receiving aspirin alone, while an increased rate of severe bleeding was noted in patients receiving ticagrelor 1 day before or just before surgery [15–18]. Hansson et al. [19] reported data from the SWEDEHEART registry on CABG-related bleeding complications in ACS patients on dual anti-platelet therapy (DAPT). The authors demonstrated that in contrast to clopidogrel, discontinuation of ticagrelor 3 days before surgery, as opposed to 5 days, did not increase the incidence of major bleeding [20]. In the PLATO trial, ticagrelor treatment was recommended to be withheld for 1–3 days before CABG [21, 22]. Current guidelines recommend that dual antiplatelet therapy be withheld at least 3 days prior to surgery [23, 24].

Management of patients treated with ticagrelor is challenging, and currently there is no effective therapeutic option. The aim of the present study was to investigate the safety of cardiac surgery when ticagrelor combined with acetylsalicylic acid (ASA) were continued to 1 day before surgery compared to ASA alone.

## Methods

A retrospective, observational study was performed to evaluate perioperative bleeding complications associated with recent Ticagrelor therapy in patients who underwent CABG. In total 161 consecutive patients underwent isolated CABG or CABG/Valvular procedures in the Cardiovascular Department at Poriya Medical Center, Tiberias, Israel. Data was collected from the departmental clinical registry. The registry and study were approved by our hospital ethics review board. Patients were divided into two groups: patients preoperatively receiving ticagrelor + acetylsalicylic acid (ASA) (group A, *n* = 55) or ASA alone (group B, *N* = 106) until cardiac surgery.

Data on peri-operative events from admission to discharge were collected. We assessed postoperative blood loss volume during the first and second postoperative days, which was defined by the total chest tube drainage. The indications for surgical re-exploration were chest blood loss > 400 ml over the first hour, > 300 ml for two consecutive hours or > 200 ml for four consecutive hours. The primary end point was defined as the composite of red blood cells (RBCs) transfusion ≥1000 ml, chest drainage ≥2000 ml and bleeding requiring surgical re-exploration [16]. We also compared the incidence and number of allogeneic blood products (plasma and platelets) transfused during the index hospital stay.

Surgical technique and postoperative management were standardized for all patients. All patients underwent on-pump CABG. A median sternotomy followed by cardiopulmonary bypass was instituted with the use of ascending aortic cannulation and two-stage venous cannulation of the right atrium. The membrane oxygenator was primed with 1000 ml of Hartmann’s crystalloid, 500 ml gelofusine, 0.5 g/kg mannitol, 7 ml of 10% calcium gluconate and 6000 IU heparin. Alpha-stat pH management was used, and the systemic temperature was kept between 32 and 36 °C. Myocardial protection was achieved with intermittent hyperkalaemic cold blood cardioplegia.

At the end of surgery, patients were transferred to the intensive care unit. Values of prothrombin time, activated partial thromboplastin time and international normalized ratio of > 1.5 times control were corrected with fresh frozen plasma. A platelet count < 60,000/μl was an indicator for platelet transfusion. A haemoglobin < 8 g/dl was the threshold for transfusion of red blood cells. According to unit policy, patients received ASA 300 mg suppository per gastric tube within 6 h after surgery; in case of bleedings, antiplatelet treatment was delayed to the day after.

### Statistical analysis

Based on the ~ 20% incidence of major bleeding complications in patients treated with dual antiplatelets stopped after 72 h prior to surgery [16], we needed a patient sample size of 93 to detect a significant doubling of its incidence (type I error *p* < 0.05, type II error < 0.1).

Categorical variables were expressed as percentages and continuous variables as means ± standard deviations. Subgroups were compared using the Wilcoxon-Mann-Whitney non-parametric test or independent t-test as appropriate. Chi-Square analysis was used for categorical variables. Statistical significance was defined as a *p* value < 0.05. Multivariable logistic regression analysis was performed to assess the association between age, smoking, number of coronary stents in situ, preoperative ACEi/ARBs, hemoglobin and use of ticagrelor with major bleeding complications. Statistical analyses were performed using MedCalc Statistical Software version 15.6.1 (MedCalc Software bvba, Ostend, Belgium).

## Results

In total, we included 161 patients who underwent CABG in our hospital. The patients were divided into two groups: patients preoperatively receiving acetylsalicylic alone (group A, *N* = 106) and patients receiving ticagrelor+ASA (group B, *n* = 55). All the 55 patients in group B received Ticagrelor, with the last dose in both groups given the day before surgery. There were no significant differences between the two groups in patients who received aspirin or anticoagulant therapy (Table 1).

**Table 1:** Preoperative baseline data of study group and control group

	Group A ASA alone %(*n* = 106)	Group B Ticagrelor +ASA %(*n* = 55)	*p*-Value
Age (years)	64 ± 8	61 ± 10	0.034
Sex, Male (n,%)	87 (92)	96 (53)	0.092
**Risk profile**			
Diabetes Mellitus	42 (45)	47 (26)	0.622
Hypertension	71 (75)	76 (42)	0.461
Hyperlipidemia	75 (80)	76 (42)	0.850
Smoking, current	38 (40)	54 (30)	0.002
Smoking, past	12 (13)	5 (3)	0.267
Obesity	16 (17)	25 (15)	0.207
**Previous CAD**	36 (38)	40 (22)	0.733
Coronary stents in situ	26 (28)	51 (25)	0.003
Paroxysmal atrial fibrillation	7 (7)	2 (1)	0.054
Renal Failure	3 (3)	5 (3)	0.414
Congestive heart failure	18 (19)	13 (7)	0.500
Peripheral vascular disease	7 (7)	2 (1)	0.265
Cerebrovascular accident	6 (6)	9 (5)	0.514
COPD	6 (6)	5 (2)	0.716
**Medications**			
Aspirin	92 (97)	98(54)	0.137
Anticoagulant therapy (VKA/DOACs)	8 (9)	2 (1)	0.254
ACEi/ARB	48 (51)	61 (38)	0.002
β Blockers	72 (76)	85 (47)	0.085
Calcium Channel Blockers	26 (28)	27 (15)	0.854

*CAD* Coronary artery disease, *ASA* acetylsalicylic acid, *COPD* chronic obstructive pulmonary disease, *ACE* angiotensin-converting enzyme, *VKA* Vitamin K antagonists, *DOACs* Direct oral anticoagulants

Baseline data are presented in Table 1. Baseline parameters showed no significant difference between the two groups. There were no differences in sex, most cardiac risk factors and preoperative medications. Notably, patients on pre-operative anticoagulants were not treated by aspirin. Patients in Groups B were ~ 5 years younger than patients in group A. Current smoking was also more prevalent in Group B. Patients in group B had twice as many previous coronary interventions with coronary stents in situ as patients in group A. A greater percentage of patients in group B used an Angiotensin Converting Enzyme Inhibitor (ACEi) or Angiotensin Receptor Blocker (ARB) demonstrated in Table 1. Both groups had the same euro and syntax score. Pre-operative LV systolic function was similar among both groups. Patients on dual anti-platelet therapy (group B) had 0.5 g/dl significantly lower pre-operative hemoglobin level compared to patients on ASA alone (group A), (*P* = 0.025, Table 2).

**Table 2:** Pre-operative status

	Group A ASA alone *n* = 106	Group B Ticagrelor +ASA *n* = 55	*p*-Value
Euroscore	2% ± 2	3% ± 2	0.729
Syntax Score	28.7 ± 9.8	28.9 ± 8.8	0.602
LV ejection fraction (%)	54 ± 11	55 ± 11	0.884
Hemoglobin (gr/dl)	11.3 ± 1.4	10.8 ± 1.5	0.025

*EuroSCORE* European System for Cardiac Operative Risk Evaluation, *SYNTAX* score is a grading system that evaluates the complexity and prognosis of patients undergoing percutaneous coronary intervention (PCI), *LVEF* left ventricular ejection fraction, *NYHA* New York Heart Association

The two groups were balanced with regard to surgery characteristics (Table 3). All patients in both groups received the same treatment protocol, perioperative hemostasis was maintained and any intraoperative bleeding was adequately controlled by standard surgical techniques.

**Table 3:** Surgery characteristics

	Group A ASA alone %(*n* = 106)	Group B Ticagrelor +ASA %(*n* = 55)	*p*-Value
Coronary Artery Bypass graft	99 (105)	100 (55)	NS
LIMA	98 (104)	100 (55)	NS
No. of Grafts	2.3 ± 0.9	2.5 ± 0.8	0.345
Concomitant valve surgery (n, %)	18 (19)	2 (1)	0.001
Aortic valve	11 (12)	0 (0)	0.01
Mitral valve	8 (9)	2 (1)	NS
Tricuspid valve	1 (2)	0 (0)	NS
Temperature (° Celsius)	36.0 ± 0.7	36.1 ± 0.7	0.732
Pump time (minutes)	105 ± 41	102 ± 36	0.250

*LIMA* left internal mammary artery

Nearly all patients received a left internal mammary arterial graft in addition to receiving 2.3 to 2.5 grafts; concomitant valvular surgery was significantly more prevalent in the ASA alone group, mainly due to increased aortic valve surgery. Mean cardiopulmonary bypass time was not different between the groups. There were no differences in the levels of troponin between the groups.

Table 4 shows the outcome data in this study. Patients in group B had a 13 h shorter stay in the intensive care unit post-operatively. However, both groups had similar mechanical ventilation time and rate of intra-aortic balloon pump usage. Length of hospital stay was similar between the two groups.

**Table 4:** Outcome data

	Group A ASA alone %(*n* = 106)	Group B Ticagrelor +ASA %(*n* = 55)	*p*-Value
Time in intensive care unit (hrs)	93 ± 70	70 ± 24	0.005
Ventilation time (hrs)	13 ± 13	15 ± 16	0.990
Intraaortic balloon pump (%, n)	2 (2)	4 (2)	0.608
Post operative discharge day	8.3 ± 8.1	7.3 ± 4.6	0.552
Lowest Hemoglobin (gr/dl)	8.6 ± 1.2	8.4 ± 0.9	0.028
Δ Hemoglobin (gr/dl)	2.7 ± 1.3	2.5 ± 1.3	0.804
Chest drainage volume (ml)			
Day 1	569 ± 393	649 ± 427	0.854
Total volume	1477 ± 1012	1454 ± 715	0.086
**Blood products infused (units)**			
Red blood packed cells	1.2 ± 1.6	1.3 ± 1.3	0.576
Fresh frozen plasma	0.5 ± 1.2	0.4 ± 1	0.112
Cryoprecipitate	0.2 ± 1.4	0.1 ± 0.8	0.420
Platelets	2.3 ± 3.8	2.4 ± 3.4	0.547
**Complications (n,%)**			
Bleeding requiring surgical re-exploration	0	0	NS
Neurologic (incl. stroke)	1 (1)	0 (0)	NS
Pneumonia	4 (4)	5 (3)	0.692
Infection (any)	5 (5)	10 (5)	0.316
Renal failure	10 (10)	2 (1)	0.099
Requiring dialysis	0	0	NS
Discharged with dialysis	0	0	NS
Atrial fibrillation	38 (40)	22 (12)	0.035
Permanent pacemaker	0 (0)	2 (1)	NS
Gastrointestinal	0	0	NS
**Primary End Point**			
(RBC > 1000 cc, drainage≥2000 cc, or Revision)	26 (28)	27 (15)	NS

Values are given as mean + SD

The primary end point composite of major bleeding complications (RBC infusion of ≥1000 ml, chest drainage ≥2000 ml or bleeding requiring surgical re-exploration) was found in just above a quarter of patients in both groups (Table 4). The incidence of major bleeding complications remained unchanged after exclusion of 20 patients that had concomitant valve surgery. Regarding blood loss, there was a small yet significantly lower hemoglobin threshold (< 8.0 (gr/dl) treated by ASA + ticagrelor compared to patients treated with ASA alone.

Hemoglobin change from baseline was similar in both groups as was the post-operative utilization of blood products (red blood cells, fresh frozen plasma, platelets and cryoprecipitate), where the transfusion triggered hemoglobin was below 8.0 (gr/dl).

Total and Post-operative day 1 pleural/pericardial chest drainage volumes were similar as well.

In multivariable logistic regression analysis that included age, smoking, number of coronary stents in situ, preoperative ACEi/ARBs, hemoglobin and use of ticagrelor, only higher hemoglobin levels was found to be associated with reduction of major bleeding complications (HR = 0.73, CI 0.55–0.97, *p* = 0.0246).

Non-bleeding post-surgical complications were similar in both groups except for atrial fibrillation, the prevalence of which was significantly lower in group B. None of the patients had stent thrombosis. No patients in either group underwent surgical re-exploration for a major bleeding complication.

Subgroup analysis of patients with CABG surgery only, who did not undergo valvular surgery, confirmed that the primary end-point composite of major bleeding complications remained similar between groups A and B [25.9% (*n* = 22) vs 25.9% (*n* = 14), *p* = 0.995]. Chest drainage volume on day 1 (561 ± 397 ml vs 642 ± 428 ml, *p* = 0.26) total drainage volume (1520 ± 1049 vs. 1437 ± 712, *p* = 0.58), and blood products infusion were not different between the two groups.

## Discussion

Many patients needing coronary surgical revascularization have significant left main disease or multi-vessel coronary disease. DAPT is usually prescribed in these patients after diagnostic coronary angiography, PCI and/or stent implantation pending surgical revascularization [25]. Yet, the optimal discontinuation time of P2Y12 antagonists prior to CABG is poorly defined. Current guidelines recommend holding the drug for at least 3 days for ticagrelor or 7 days for prasugrel prior to surgery [9, 23, 24]. However, this may not be a realistic option in every case, as discontinuation of DAPT may actually increase the risk of stent thrombosis complicating the perioperative course. Several studies have shown that administration of ticagrelor prior to cardiac surgery, especially in combination with ASA, may increase the risk of postoperative bleeding, resulting in an increased administration of blood products, re-thoracotomies and lengthening of post-operative hospital stay [15–18]. In a sub-study of the PLATO trial, ticagrelor was stopped 24–72 h prior to CABG surgery [26]. The authors demonstrated that the total and cardiovascular mortality decreased without an increased risk of bleeding. However another study where ticagrelor continued until the last day prior to surgery demonstrated a clear trend towards a higher incidence of major bleeding [27].

A recent systematic review [28] summarized the updated guidelines for perioperative DAPT treatment in patients who are candidates for CABG surgery. The guidelines discussed provide differing recommendations, which may lead to a lack of standardization in clinical practice. Overall, the authors classify their recommendations based on the urgency of surgery performed.

For elective patients, most guidelines recommend that aspirin be continued while clopidogrel and ticagrelor should be discontinued at least 5 days before surgery, and prasagruel 1 week prior. A few recent sources advise stopping ticagrelor 3 days prior to surgery [29–31], which was supported by a patient cohort that demonstrated no increase in major bleeding complications [19]. In regards to the urgent CABG, various guidelines recommend discontinuing dual antiplatelet therapy between 24 and 72 h prior to surgery [5, 28, 32–35]. However, ESC 2015 [4] and TSC 2018 [30] propose that the urgent CABG could be performed regardless of their continuation. Therefore, the consequences of delaying an urgent surgery due to an increased risk of bleeding compared to early discontinuation of DAPT is still under debate [28]. Following CABG, antiplatelet therapy is recommended in post-stent patients to help prevent graft occlusion and stent thrombosis for 12 months or 1–12 months depending on risk of thrombosis [4, 5, 29–31]. Overall, due to the discrepancy between guideline recommendations, more evidence is required to standardize the perioperative management of DAPT in CABG patients.

In this study, we observed whether patients undergoing CABG under continued preoperative use of ticagrelor+ASA differs from patients under ASA alone. Patients treated with ASA + ticagrelor were 3 years younger, were more currently smoking and had twice as many had coronary stents in situ compared to patients treated with ASA alone prior to surgery, probably establishing the reason for DAPT in these patients. However, pre-operative left ventricular systolic function and risk scores were comparable in both groups, as were the surgical interventions added to CABG.

Since concomitant CABG and valvular surgery may pose a higher risk of bleeding, we performed a subgroup analysis of patients with CABG surgery alone who did not undergo valvular surgery. The subgroup analysis confirmed that the primary end-point composite of major bleeding complications, chest drainage volumes and the infused blood products were not significantly different.

Post-operatively, both groups showed similar bleeding parameters, including maximal hemoglobin drop, utilization of blood products, and day 1 and total chest drainage volumes. Complication rates were also similar and no patients had a stent thrombosis.

This study shows that among patients initially treated for ACS with antiplatelets that subsequently underwent CABG, ticagrelor+ASA compared with ASA alone was not associated with risk of CABG-related bleeding. This observation suggests that it may be safe to operate without discontinuation of ticagrelor for at least 5 days prior to surgery as currently recommended in guidelines; however, future studies are needed to further evaluate the risk of CABG-related bleeding under recent ticagrelor therapy.

### Study limitations

This was a single center retrospective study in a special setting of a fully combined cardiology-cardiovascular surgery service, which may have introduced treatment bias, likely affecting outcomes. Future randomized controlled studies should be performed to confirm our findings. Our surgeons were not blinded to pre-operative treatment and probably anticipated and reacted to bleeding during surgery, which may have led to improved hemostatic treatment in the DAPT group. This practice of meticulous hemostasis in DAPT treated patients has probably contributed to the comparable outcomes in both groups. Additionally, genetic polymorphisms may affect bleeding risk. However, since this is a retrospective study, genotype analysis was not performed. Future studies including genotypic analysis should be conducted to investigate how gene polymorphisms play a role in bleeding complications.

## Conclusion

Continuing ticagrelor until surgery did not increase the risk of bleeding complications after CABG compared with patients on ASA alone. The overall risk of CABG related bleeding complications was the same in ticagrelor + ASA group compared ASA alone in this observational study. Although patients on dual therapy are known to be at increased risk for perioperative bleeding complications, continuing ticagrelor until surgery may be safe. Future studies are warranted to investigate the risk of CABG-related bleeding under recent ticagrelor therapy.

## Data Availability

Please contact the authors for data requests.

## Abbreviations

ASA: Acetylsalicylic acid
ACS: Acute coronary syndrome
CABG: Coronary artery bypass graft (surgery)
CAD: Coronary artery disease
COPD: Chronic obstructive pulmonary disease
DAPT: Dual anti platelet therapy
